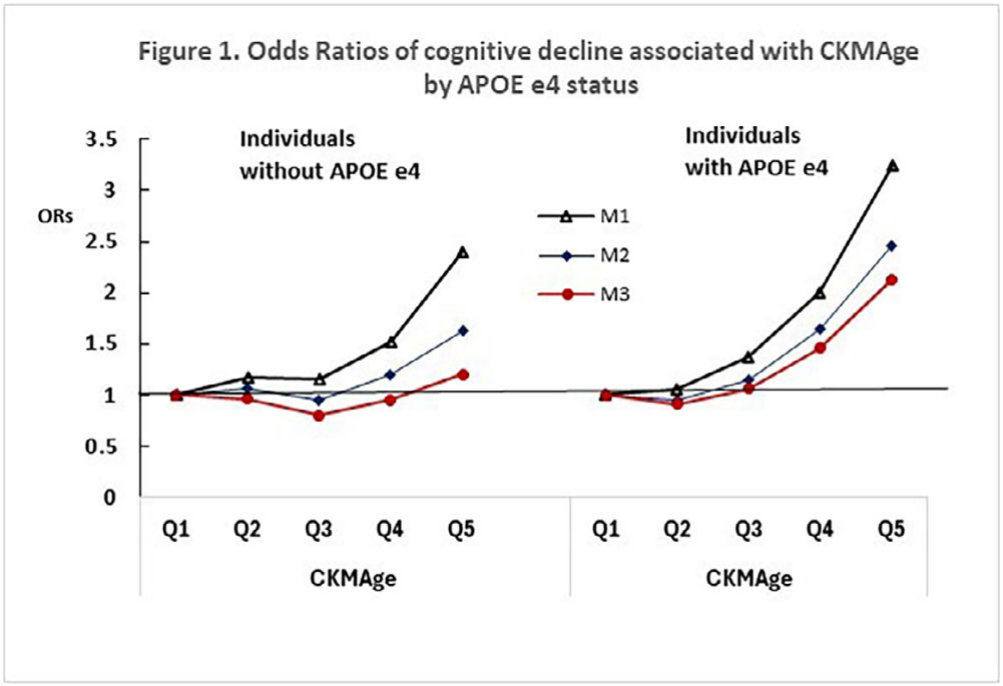# Association of CKM Phenotypic Age Acceleration and APOE Genotype with Risk of Cognitive Decline in U.S. Adults Aged 50 and Over

**DOI:** 10.1002/alz70856_102835

**Published:** 2025-12-24

**Authors:** Longjian Liu, Jintong Hou, Yvonne L Michael

**Affiliations:** ^1^ Drexel University Dornsife School of Public Health, Philadelphia, PA, USA; ^2^ Drexel University Dornsife School of Public Health, Philadephia, PA, USA

## Abstract

**Background:**

The association of cardio‐kidney‐metabolic (CKM) syndrome with cognitive decline and APOE genotype remains unclear. This study aimed to investigate the association of CKM phenotypic age (CKMAge) and cognitive decline, considering the effect modification by Apolipoprotein E (APOE) genotype in U.S. adults.

**Methods:**

A nationally representative cohort of 13,222 adults aged ≥50 years in the 2008 U.S. Health and Retirement Study was analyzed. CKMAge, a novel biological aging indicator, was developed using four CKM markers (hypertension, systolic BP, serum Cystatin C, and hemoglobin A1c) and chronological age. Cognitive function was assessed using a validated 27‐point scale, with cognitive decline defined as the lowest quantile (Q1). APOE alleles (e2‐e4) were genotyped to evaluate the genetic risk. Logistic regression models estimated odds ratios (OR) for cognitive decline associated with CKMAge and APOE genotype. Area under the curves (AUC‐ROC) analyses assessed classification performance for cognitive decline.

**Results:**

We observed a linear trend with participants in the highest CKMAge quantile (Q5) having the highest rate of cognitive decline (38.5%), followed by those in Q4 (23.8%), Q3 (16.3%), Q2 (13.4%), and Q1 (9.8%) (*p* <0.001). Each 5‐year increase in CKMAge was associated with a 21% higher risk of cognitive decline (OR: 1.21, 95%CI: 1.15‐1.27) in a model adjusted for key covariates. In multivariate logistic models, this association attenuated by 10.7% after further adjusting for diabetes, cardiovascular disease (CVD), and renal dysfunction (Figure 1, Model 3). A significant interactive effect between CKMAge and APOE e4 was observed (OR: 1.35, 95%CI: 1.11‐1.65). CKMAge correctly classified 66.5% of the cases (AUC: 0.665, 95% CI: 0.654–0.677), while APOE genotype alone classified 52.0% (AUC: 0.520, 95% CI: 0.511–0.530). The combination of CKMAge and APOE genotype in a fully adjusted model improved classification accuracy to 72.1% (AUC: 0.721, 95% CI: 0.710‐0.731).

**Conclusions:**

CKM phenotypic age significantly contributes to cognitive decline in addition to the impact of APOE e4. These findings offer new insights into the mechanisms linking CKM phenotypic age and cognitive decline, paving the way for the development of novel therapeutic and preventive strategies for cognitive impairment.